# Climate, landscape, and life history jointly predict multidecadal community mosquito phenology

**DOI:** 10.1038/s41598-023-30751-4

**Published:** 2023-03-08

**Authors:** Lindsay P. Campbell, Mohamed F. Sallam, Amely M. Bauer, Yasmin Tavares, Robert P. Guralnick

**Affiliations:** 1 Florida Medical Entomology Laboratory, Department of Entomology and Nematology, IFAS, University of Florida, 200 9th St SE, Vero Beach, FL 32962 USA; 2 Preventative Medicine Biostatistics Department, Uniformed Services University of the Health Sciences, Bethesda, MD 20814 USA; 3 Department of Natural History, Florida Museum of Natural History, Dickinson Hall, University of Florida, Gainesville, FL 32611 USA

**Keywords:** Ecology, Ecology, Environmental sciences

## Abstract

Phenology of adult host-seeking female mosquitoes is a critical component for understanding potential for vector-borne pathogen maintenance and amplification in the natural environment. Despite this importance, long-term multi-species investigations of mosquito phenologies across environments and differing species’ life history traits are rare. Here we leverage long-term mosquito control district monitoring data to characterize annual phenologies of 7 host-seeking female mosquito species over a 20-year time period in suburban Illinois, USA. We also assembled data on landscape context, categorized into low and medium development, climate variables including precipitation, temperature and humidity, and key life history traits, i.e. overwintering stage and Spring–Summer versus Summer–mid-Fallseason fliers. We then fit linear mixed models separately for adult onset, peak abundances, and flight termination with landscape, climate and trait variables as predictors with species as a random effect. Model results supported some expectations, including warmer spring temperatures leading to earlier onset, warmer temperatures and lower humidity leading to earlier peak abundances, and warmer and wetter fall conditions leading to later termination. However, we also found sometimes complex interactions and responses contrary to our predictions. For example, temperature had generally weak support on its own, impacting onset and peak abundance timing; rather temperature has interacting effects with humidity or precipitation. We also found higher spring precipitation, especially in low development contexts, generally delayed adult onset, counter to expectations. These results emphasize the need to consider how traits, landscape and climatic factors all interact to determine mosquito phenology, when planning management strategies for vector control and public health protection.

## Introduction

The timing of terrestrial insect adult seasonal emergences, peaks and termination, i.e. key intervals in their phenologies, critically impacts ecosystems and their services, and is one of the most sensitive indicators of the effects of global environmental change^1^. Although multiple studies have focused on temporal trends in insect phenology, ranging from pollinator species^2,3^ to crop pests^4^, few studies have investigated mosquito phenologies over multidecadal time scales, despite the global importance of mosquitoes to human and veterinary health^5^. This gap is especially important to close because mosquito phenology of adult host-seeking females is a critical component for understanding potential for pathogen maintenance and amplification in the natural environment^6,7^. In addition, correlating environmental variables and life history traits to key phenological intervals (i.e. onset, timing of peak abundances, and flight termination) can provide useful information that be informative to planning abatement and control efforts and toward prediction under current and future environmental conditions.

Mosquitoes are ectotherms and require aquatic habitats for multiple life stages^8^. Thus, their seasonal phenology should link closely to environmental conditions, providing a means to predict the timing and distribution of abundances under current and future environmental change^9^. In temperate regions, winter dormancy is a particularly crucial life-history strategy for timing life cycles to avoid unfavorable conditions, and thus increase survivorship^7^. The timing of entering and exiting dormancy is in part determined by environmental signaling, and often linked to climatic events such as the first hard frost and decreased photoperiod for initiating adult flight termination and dormancy^10,11^, while increasing day length and accumulation of heat e.g., growing degree days^12^ reinitiates egg or larval development or adult activity. While extrinsic, abiotic factors are fundamental, intrinsic characteristics related to long-term selective contexts can also lead to significant variation among species in both timing of when dormancy begins or ends, and what conditions are faced across different life stages^13^. For example, winter diapause stage (e.g., egg, larval or adult) may directly impact adult phenology responses, since each stage has different physiological tolerances and developmental rates in late winter or spring conditions^14^. As well, whether a species flies earlier or later in the season may also impact responsiveness to different environmental cues as has been shown in other work^15^.

A final, and potentially particularly important driver of adult phenology relates to landscape and how it has been altered. While climatic conditions are often considered the key environmental driver of adult phenology, it has been shown that different landscape factors can also impact phenology^16^. In particular, more developed landscapes may provide more microhabitats, and overall milder winters, compared to wildlands, which may shift adult mosquito emergence earlier and termination later^16,17^. Landscape impacts on phenology may also vary contextually in different climatic conditions^18^, but such context-dependence has also not been examined in insects generally, and mosquitoes in particular.

Key genetic and physiological mechanisms underlying seasonal mosquito abundance have started to be elucidated^19^, but far less common are long-term, multi-species investigations of mosquito phenologies across heterogeneous environments. Further, while the proximate cues driving especially adult emergence and termination have long been proposed (e.g., photoperiod, temperature)^13^, studies that determine how multiple environmental drivers, including landscape, can interact to determine key phenological events are far less common. Studies addressing long-term phenology trends using multiple species found in different climate and landscape contexts, and with differing life-histories, are missing from the literature.

Recent digitization efforts of routine mosquito control trap surveillance data in combination with Earth systems monitoring data provide new resources to address outstanding questions about how phenology is shifting in the face of environmental change^20^. These data resources often capture very fine grain spatial and temporal sampling over relatively broad regions and over long periods of time. Some mosquito surveillance extends over decades, using consistent trapping methods at the same set of sites, with consistent start and stop times and cadence of sampling. These rich datasets can be coupled to increasingly high-quality retrospective climatic data, along with land cover assessments that are updated every few years^21^. These data resources, when coupled with statistical modeling frameworks, can uncover the strength of drivers of adult phenology, as well as provide a means to understand trends and subtrends in phenology change, all of which can feed into better forecasting of mosquito-borne transmission hazard into the future.

Here we showcase the utility of long-term mosquito monitoring and surveillance to characterize the annual phenologies of Spring-Summers eason and Summer-mid-Fall season host-seeking female mosquito species over a 20-year time period across 18 trap locations in Illinois, USA, and to quantify how landscape, climate, life history traits, and their interactions drive adult emergence, peak abundances, and adult flight termination. Based on previous work, we expected to find: (1) earlier onsets during years with higher average spring temperatures, precipitation, and humidity; (2) later termination in higher summer precipitation and warmer summer temperatures; (3), earlier peak phenology (when abundances are highest) when temperatures are warmer, and precipitation and humidity low. We also expected to find earlier onset for mosquitoes found in locations surrounded by greater percentages of developed land cover within foraging/flight ranges. Finally, we anticipated that land cover context may drive phenologies, especially given urban heat island effects, and that this effect is stronger in colder years. These climate -landscape interactions may also be further conditioned by key traits i.e., overwintering stage and seasonal flight timing.

## Results

### Key species and species traits assembly

Seven species across four genera contained sufficient data to calculate phenometrics (Table 1). Overwintering traits for individual species included egg and adult, with all three *Aedes* species classified as overwintering as eggs and the remaining species classified as adult. Although *Aedes triseriatus* can overwinter as larvae if tree holes remain unfrozen, we classified the overwintering stage as “egg” given the northern latitude of the study area and associated low winter temperatures in this region^22^. Spring–Summer and Summer-mid-Fall season species, classified by peak abundances occurring prior to or later than Julian day 216 (August 4th, perpetual; August 5th leap year) resulted in five Spring–Summer season species and two Summer-mid-Fall season species; species with bimodal peak abundances were classified as “Spring–Summer”. We chose Julian day 216 based on this date occurring at the middle point of the sampling season included in our analyses, however species flight curves (Supplementary Figs. 1, 2) demonstrate that this date is reasonable given the flight curve distributions.

**Table 1:** Species included in analyses with trait classifications including overwintering stage, and timing of peak abundance in adult stage with “Spring–Summer occurring prior to August 4th each year and “Summer–mid-Fall” occurring after August 4th; bimodal flight patterns are classified as “Spring–Summer.”

Species	Trait overwinter	Trait Spring–Summer/Summer–mid-Fall	Source
*Aedes triseriatus*	Eggs	Spring–Summer	https://wrbu.si.edu/vectorspecies/mosquitoes/triseriatus
*Aedes trivittatus*	Eggs	Spring–Summer	https://wrbu.si.edu/vectorspecies/mosquitoes/trivittatus
*Aedes vexans s.l*	Eggs	Spring–Summer	https://wrbu.si.edu/vectorspecies/mosquitoes/vexans
*Uranotaenia sapphirina*	Adult	Summer–mid-Fall	https://vectorbio.rutgers.edu/outreach/species/sapp.htm
*Culex territans*	Adult	Summer–mid-Fall	https://vectorbio.rutgers.edu/outreach/species/terr.htm
*Culex pipiens s.l*	Adult	Spring–Summer	https://wrbu.si.edu/vectorspecies/mosquitoes/pipiens
*Anopheles punctipennis*	Adult	Spring–Summer	https://academic.oup.com/jme/article/54/5/1344/3789108

**Figure 1:**
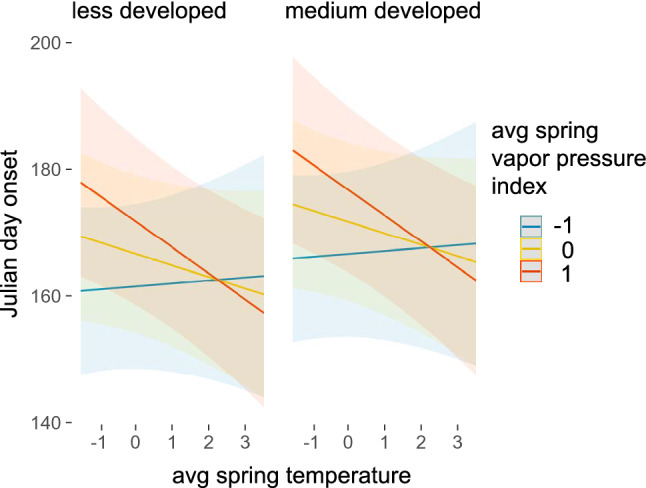
The effect of temperature does not vary across landscapes, and leads to earlier onset in drier conditions (e.g., higher spring vapor pressure deficit) but has minimal impact on phenology in wetter conditions. These plots also demonstrate a weak effect of later onset in medium developed landscapes. Plots were generated using the ‘plot_model’ function in the *sjPlot* package and functions in *ggplot2* package in R.

**Figure 2:**
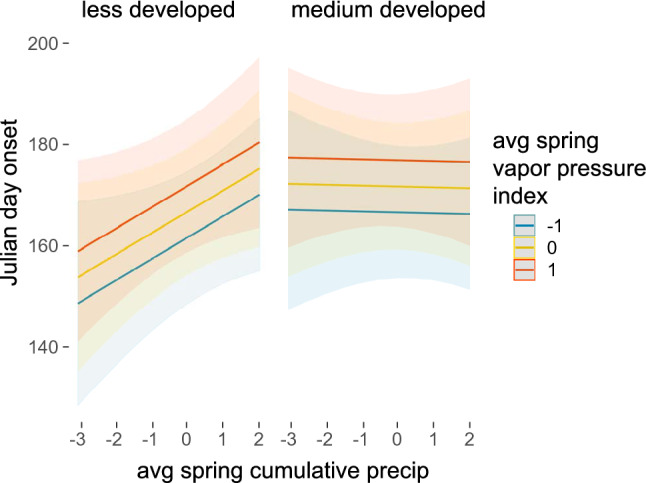
More cumulative precipitation leads to much later onset in less developed regions, but has no impact on phenology in more developed ones. In all cases, higher spring vapor pressure deficit (drier conditions) leads to later onset. Plots were generated using the ‘plot_model’ function in the *sjPlot* package and functions in *ggplot2* package in R.

Overall results for onset phenology suggest our best model after stepwise variable reduction is informative (marginal and conditional R^2^ values were 0.483 and 0.59, respectively). Model results for onset showed that higher average temperature in spring–summer leads to earlier onset, but this relationship is strongest when vapor pressure deficit (a proxy for humidity) is highest. Thus, in highest humidity conditions, temperature no longer strongly drives phenology (Fig. 1). A surprising result is that both lower humidity (expressed here as higher vapor pressure deficit) and higher cumulative precipitation lead to later onset of phenology, although precipitation (but not vapor pressure deficit) impacts are much weaker in more urbanized, developed areas compared to less developed ones (Fig. 2). In sum, higher precipitation especially in low development regions, and drier overall conditions act to delay onsets. It may seem counterintuitive that precipitation and humidity show opposite effects on onset phenology, but as we discuss below, focusing on average values over a season means that a season could have punctuated heavy rainfall but still be drier than usual. We finally note one surprising impact of landscape on onset timing of mosquitoes—onset is later in all species in more developed areas, rather than earlier, as we expected, and Summer-Fall season species are particularly impacted towards later onset (Supplementary Table 1).

**Figure 3:**
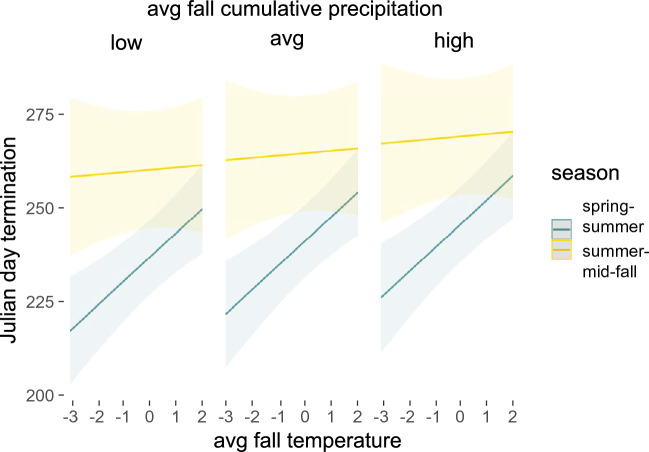
Increasing average late Summer-early Fall temperatures lead to much later termination in Spring–Summer flying species, but this impact is much weaker for Summer-mid-Fall flying ones. More precipitation also leads to later termination but the strength of this predictor is not conditioned on flight timing. Plots were generated using the ‘plot_model’ function in the *sjPlot* package and functions in *ggplot2* package in R.

Model results for termination supported our expectations; higher average cumulative precipitation and average fall temperatures lead to later termination dates. However, a significant negative interaction of average fall temperature with Summer-mid-Fall season species demonstrated that warmer temperatures were not a strong predictor for Summer-mid-Fall season species termination; as temperatures increased, termination dates increased only slightly, especially compared with the very strong effect of temperature on termination in Spring–Summer season species (Fig. 3). Marginal and conditional R^2^ values for the best model after stepwise variable reduction were 0.303 and 0.499.

**Figure 4:**
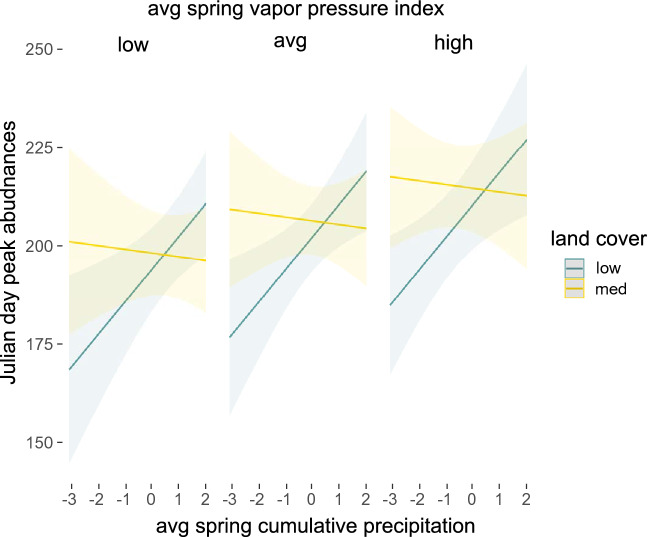
Average spring cumulative precipitation leads to much later peak abundances in areas classified as low development, but slightly earlier peaks in medium developed areas. Drier conditions (higher vapor pressure deficit) lead to later peak abundances independent of land cover type. Plots were generated using the ‘plot_model’ function in the *sjPlot* package and functions in *ggplot2* package in R.

Model results for peak abundances indicated that average spring cumulative precipitation and mean vapor pressure deficit values had a positive effect on peak abundance. Temperature on its own did not impact peak phenology timing, but it strongly interacts with cumulative precipitation such that warm and dry conditions lead to much earlier peak abundances, while warm and wet conditions lead to much later peaks (Fig. 4). Surprisingly, the effect of precipitation is itself different across low and medium developed landscapes, with peak abundances showing weakly negative responses to precipitation in medium developed areas and strongly positive responses in low developed ones. Conditional R^2^ values (0.391) were lower for the final peak abundance model compared to onset and termination models. Full model results are available in Supplementary Table 1 and conditional values for intercept terms for species-level random effects are available in Supplementary Table 2.

## Discussion

Investigations of mosquito phenology most often focus on short time periods and the effects of single environmental variables on individual species. Here, we leveraged routine mosquito control surveillance data over a 20-year time period and showed that climate, landscape, life history traits, and their interactions have strong and complicated effects on multispecies mosquito phenology in temperate climate conditions in suburban Chicago. These results emphasize the need to identify and account for variation in mosquito phenology across heterogeneous environment and life history traits when planning management strategies for vector control and public health protection, even when working across smaller geographic areas.

Our results confirm the importance of temperature as a driver of mosquito phenology, but its impact is both conditional on other climatic and landscape drivers and its importance varies across phenology stages. Somewhat surprisingly, temperature has the strongest direct impact on mosquito flight termination timing, particularly for Spring–Summer season species, rather than onset or timing of peak abundances. Instead, onset and peak phenology is driven much more strongly by humidity, precipitation and landscape composition. This weaker effect of temperature on onset, where it is conditionally important only during drier years, is atypical for many insect groups^15^ but likely reflects the strong requirements for available aquatic habitats needed for larval development^8^.

We also expected precipitation to be a significant driver of phenology, given that higher average precipitation is often associated with an increased number of aquatic habitats supporting immature mosquito development^8^. Given this, we expected precipitation to drive earlier onset, and so were surprised to find that higher spring precipitation along with overall drier conditions instead delayed adult onset, especially in low development landscapes. While complex, a pattern of greater precipitation delaying onset has been observed across multivoltine insect species^15,23^. It may be that the averaged seasonal climate summaries used here do not capture sometimes rapid intraseasonal variations, which could contribute to our results. For example, high average precipitation coupled with overall drier conditions may indicate punctuated or intermittent heavy rainfall events during a drier season, which can result in flushing of immature mosquito habitats and delay onset. Flushing effects have been observed previously in *Culex* mosquito abundances from a multiyear study in New Jersey, USA, which found that overall precipitation increased adult mosquito abundances, however episodes of heavy rainfall preceding trap collections reduced counts^24^. Heavy rainfall followed by drier conditions could also impact the survival rate of adult mosquito populations due to desiccation, also delaying onset. Finally, significant early season precipitation may fall as snow, delaying onset of spring conditions. The strong effect of temperature and precipitation coupled to produce early or mid-spring poor conditions can be smoothed if there are late season warm spells when time averaging over a season. One key next step in work on spring dynamics is to utilize finer temporal-grain climatic data to detect if, for example, particularly cold and wet early springs are strong drivers of delayed adult onset.

Landscape context often had strong impacts on mosquito phenology but not always in the directions predicted and often via conditional effects with climate and traits. When precipitation is low, less developed landscapes had much earlier overall timing of mosquito emergence than in more developed areas, but this strong effect disappears when precipitation is higher. We find no evidence of earlier emergence driven by milder conditions in more built environments e.g., an urban heat effect. We also found that while warmer and wetter conditions delayed peak abundances in less developed landscapes, precipitation was not a factor in more urbanized areas. In this study, more urbanized study sites consisted of medium developed landscapes characterized by residential areas with mixed vegetation and built environments^21^ which contain extensive storm water drainage systems that include catch basins that provide larval habitats and adult resting/overwintering habitats for some mosquito species^25,26^. These habitats can provide shelter and help mitigate effects of high precipitation and provide protection against desiccation under drier environmental conditions^27,28^. While further investigation is needed of intraseasonal variation in climate conditions, including the timing and magnitude of unusually high precipitation events, catch basin environments may mitigate the effects of heavy precipitation on delayed mosquito onset and on peak abundance phenology in managed landscapes compared to more natural landscapes.

Although air humidity is an important factor for mosquito survival, we also found that higher spring temperatures coupled with drier humidity conditions advanced adult onset, and as humidity increased, the effect of temperature on onset decreased across both less developed and medium developed landscapes. In medium developed landscapes, this finding may reflect the emergence of adult mosquito populations from humid catch basins and flood drains, where they overwinter as adults and can harbor during resting periods^26,29^. In less developed landscapes, annual spring snowmelt associated with warmer late spring temperatures that do not necessarily correspond to higher humidity may also contribute to this result. Several species in our study, including species complexes such as *Aedes vexans s.l.*, can use temporary water bodies for initial larval development in the spring and if these water bodies are then regenerated through precipitation, multiple generations continue throughout the season^30^.

Interestingly, while warmer temperatures delayed termination, the effect of temperature was not strong for Summer–mid-Fall season species. The Summer–mid-Fall season species investigated here were *Cx. territans* and *Ur. sapphirina*. Both species bloodfeed on amphibian hosts, however *Ur. sapphirina* specialize on a broad range of annelid hosts and *Cx. territans* specializes on *Rana clamitans* frogs^31,32^. Previous work under low temperature scenarios found *Cx. territans* presence was closely synchronized to *R. clamitans* presence, that temperature and other environmental variables were not strong predictors of presence, and *Cx. territans* were able to digest blood meals at a low temperature threshold, suggesting that this species can undergo thermoregulation that facilitates functioning at low temperatures^31^. Although a similar study has not been conducted for *Ur. sapphirina*, our results corroborate earlier field and laboratory investigations for *Cx. territans* and scales over a longer time period, highlighting that additional life history traits including host specialization and host phenology could also be an important component of observed mosquito phenology^33^.

One life history trait that did not have an effect on mosquito phenology was overwintering stage. This result was surprising given the distribution of species overwintering as eggs and as adults in our study and expected differences in time between emergence from overwintering to adult host seeking behavior because of life stage. However, one possibility for this result could be the thresholds used here for calculating onset and termination (i.e. 15% and 85%). The advantage of this approach is capturing a substantial portion of the flight curve and reducing effects of unusual early or late individual emergence or termination^34^. However, this approach may miss nuanced early or late season dynamics that overwinter stage could impact.

Although our study leveraged a substantial 20-year mosquito trap surveillance data set across multiple trap locations, limitations exist. While four genera were represented in our study, phenometrics were calculated for a total of seven dominant or abundant species, which may not necessarily reflect the full phenological diversity of the mosquitoes in the study region. In addition, *Cx. pipiens* species in our study likely included *Cx. pipiens pipiens* and *Cx. pipiens molestus* mosquitoes in the same species designation, and in some cases species active in the fall that overwinter as adults may have been seeking nectar rather than a blood meal, which could have been missed in CO_2_ baited light traps. In addition, *Ae. vexans* mosquitoes may have included multiple subtypes. However, we expect that the overall effects of these limitations on our phenometric calculations were minimal. While we used an established approach to calculate phenometrics derived from multiple locations, the current method does not necessarily account for residual spatial autocorrelation when estimating values. However, the inclusion of trap locations into phenometric calculations by land cover type, rather than proximity to one another may have helped to mitigate this limitation. In addition, our choice to include general land cover types representative of anthropogenic activities may have resulted in missed nuances related to microhabitats and we limited our analyses to landscapes within a 1 km buffer distance of traps to provide general information about these anthropogenic activities. Future landscape investigations across multiple land cover types and within additional buffer distances from trap locations may reveal additional information. Despite these limitations, our models were informative for the set of species included here, particularly for onset, and we found that interactions between climate and landscape variables and between species traits and the environment were important predictors of mosquito phenology. In addition to multi-species studies, meta-analyses of individual mosquito species may provide useful information toward understanding environmental effects on mosquito phenology. Future investigations of unusual weather events, particularly precipitation events, have the potential to provide further insight into climate and landscape interactions important to mosquito phenology across species and life history traits.

## Materials and methods

### Assembling the mosquito control district data

**Figure 5:**
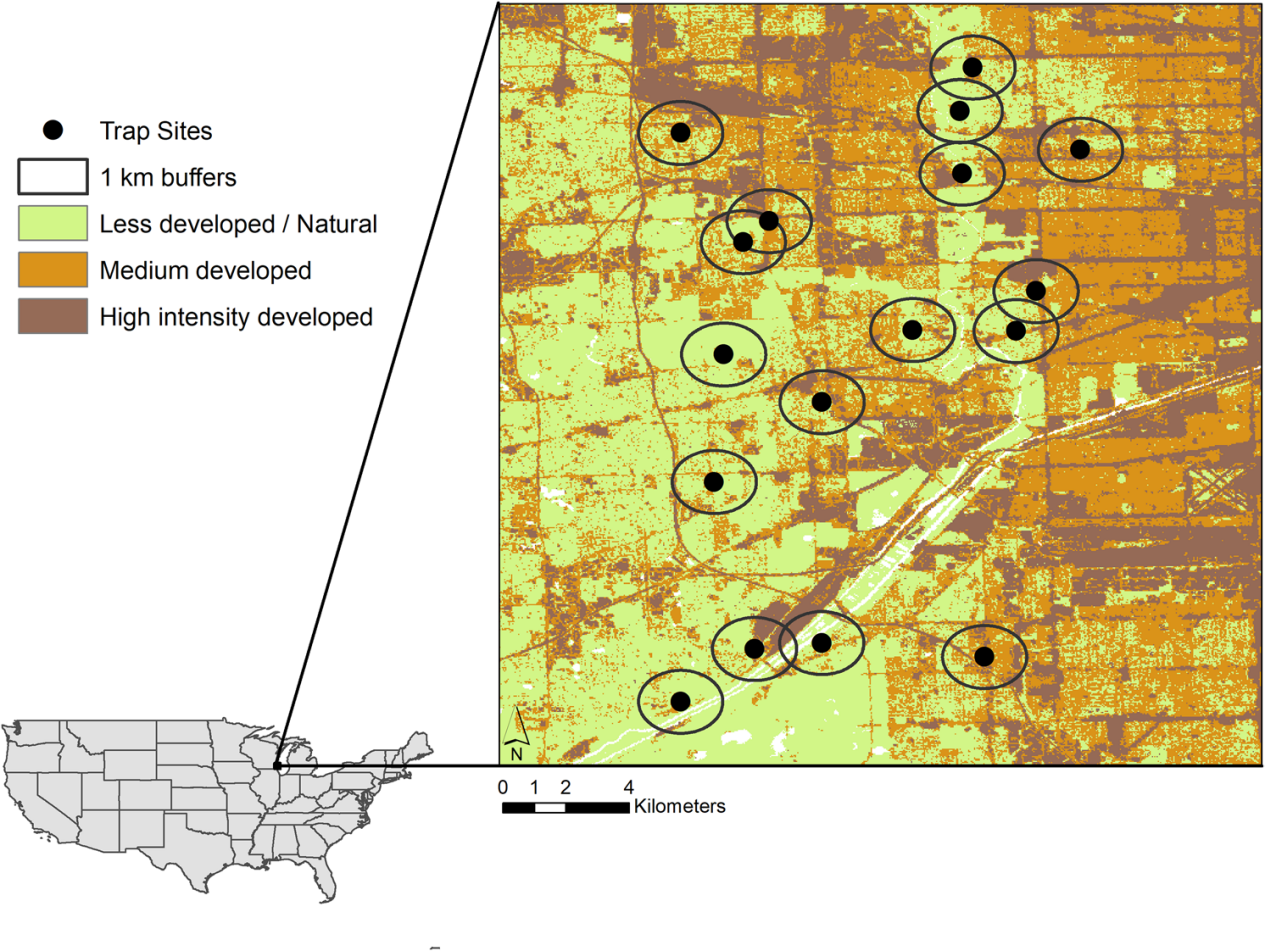
Distribution of mosquito surveillance trap sites across less developed and medium developed landscapes in suburban Chicago. Map was created using ESRI ArcMap v10.6 software program (https://www.esri.com).

We focused on Desplaines Valley Mosquito Abatement mosquito trap surveillance data collected from Cooke County, IL. This county is located in the greater Chicago Metropolitan Area, USA and includes low, medium, and high intensity developed land cover, with high intensity developed land cover in the east in the downtown metropolitan area (Fig. 5). Generally, the climate of the study sites is humid continental, as designated by the Köppen–Geiger climate classification system, and exhibits large seasonal temperature contrasts with hot summers and cold winters and local weather effects from Lake Michigan^35^.

Mosquito control surveillance trap data were downloaded from VectorBase PopBio data repository (https://vectorbase.org/popbio-map/web/)^36^. The data set included multiple fields, which are outlined in an extensive data dictionary provided through the data repository. For the purpose of this study, we included latitude and longitude coordinates of the collection site, the collection date, trap type, attractant, and an abundance value for individual species. Data were filtered to include only New Jersey light traps with visible light as the attractant between the years 2000–2020.

Once downloaded, we performed a set of quality assurance and control steps. First, we generated Location IDs for each unique set of geographic coordinates and data were reshaped to a site-by-species matrix using the ‘dplyr’ package in R^37^, with individual sites for individual collection dates serving as rows and species counts as columns. If an abundance value was NA in the site-by-species matrix, the NA value was replaced with a zero if the species had been collected across any sites on a date prior to the collection date; the only species with NAs included in the matrix was *Aedes japonicus*, which was non-native to Illinois and first recorded in 2008. We checked for timing of the start and stop of sampling per site-year combination and also checked completeness of sampling during the year for all sites (Supplementary Fig. 3).

### Assembling key landscape and climate metrics

We generated landcover metrics over the time period of the analysis (2000–2020). To do so, we downloaded USGS National Land Cover Data (NLCD) 30 m resolution data for 2001, 2004, 2006, 2008, 2011, 2013, 2016, and 2019 from the Multi-resolution Land Characteristics Consortium (MRLC https://www.mrlc.gov/data) and masked to Illinois^21^. In this study, we focused on three general land cover classes representing general anthropogenic activities surrounding mosquito trap locations. Land cover classes for each data set were reclassified to low developed (value = 1), medium developed (value = 2), and developed (value = 3). The low developed class included low intensity developed and open developed classes along with all other land cover classes except medium- intensity developed and high-intensity developed. The medium developed class included medium-intensity developed and the developed class included locations classified as high-intensity developed. A 1 km buffer was created around each unique location ID and percent land cover for each of the three classes was calculated within each buffer for each time period using the *landscapemetrics* package in R^38^ and the class with the highest percentage was assigned to the location. Because we were investigating annual phenology values from 2000 to 2020 we assigned low, medium, and high developed land cover classes at locations for each year for which the NLCD was not available (Fig. 1). In short, for each year where NLCD was not available, we chose the closest proximal year. For example, for 2002, we used the NLCD 2001 data and for 2003 we used the NLCD 2004. In this study, we only had low and medium developed landcover classes; none were in the highest development class.

Daily climate values for minimum and maximum temperature, minimum and maximum vapor pressure deficit, and total precipitation from 2000 to 2020 were acquired from 4 km resolution gridMET surface meteorological data^39^ and downloaded using the *climateR* package at individual mosquito trap locations^40^. Daily data were stratified into annual spring-early summer and summer-early fall seasons. The spring season included daily values from March 1st to June 30th during the early portion of the sampling season, and the summer-fall season included daily values between July 1st and October 31st. Annual average minimum and maximum temperature values, minimum and maximum vapor pressure deficit, and cumulative precipitation values were summarized at each location over the study period using the *tidyverse* package in R^41^. Seasonal climate summaries at individual trap locations were then grouped by year and land cover designation and average minimum and maximum temperature, average minimum and maximum vapor pressure deficit, and average cumulative precipitation were calculated across sites sharing the same land cover designation.

### Calculating mosquito flight curves and phenometrics

Phenometrics were derived for individual mosquito species representing 15% emergence, 50% relative abundance, 85% termination, and peak abundances using the *rbms* package^42^ in R. Adult 15% emergence and 85% termination metrics were chosen to reduce bias that can result from unusual early or late individual emergence or termination, while capturing a substantial portion of the flight curve^34^. The *rbms* package provides a set of wrapper scripts to calculate relative abundance indices from yearly time-series data. It does so by defining a start and end date of the monitoring season, a time step for the curve (either daily or weekly), and then uses site visit information across multiple sites to calculate a summary flight curve.

Our interest in this study are annual flight curves per species, stratified by our land cover classes (low and medium developed). Because sampling start and stop timing was highly consistent and sampling relatively complete over the season (see above and Supplemental Fig. 1), we felt confident fitting per-species flight curves across land cover types and set a minimum requirement of 2 sites sampled for a species-year-landcover group. After assembling data for fitting in *rbms*, we generated flight curves for all species-year-landcover combinations. While there are 24 species and 2 species morphological groups sampled in Illinois over the 20-year period, we could only fit phenometrics over most of the time series for the 7 most commonly sampled species. Many species are rare or sporadic in the region, making it difficult to establish a clear seasonal abundance pattern. In other cases, phenoestimates for some species could only be fit for a more limited portion of the time series, and these too we dropped to focus on species with more complete estimates over time. The end result of phenocurves for species-landscape-year combination provide an estimate of relative abundance over the season, and because the area under the curve is equal to one, can easily be used to generate our key metrics (e.g., 15% emergence, 50% median, peak, and 85% termination). All data and code used for generating and assembling phenometric data and model analyses is available on github (https://github.com/Campbell-Lab-FMEL/mosquito-community-phenology).

### Assembling key trait data and fitting phenology models

We focus on two key traits that may impact phenological responses: overwintering stage and early versus late flight timing, here designated as Spring–Summer and Summer-mid-Fall flight timing^43^. These two traits have been used in other studies and both have been shown to strongly relate to phenological responsiveness to both climate and landscape^43,44^. We determined the overwintering stage for mosquito species using literature resources (see Table 1 in “Results”). We classified species as Spring–Summer or Summer–mid-Fall flying by examining 50% flight period timing per species; species with bimodal peaks were classified as Spring–Summer. There is a clear bimodal distribution separating those species whose median flight timing is prior to early August and those whose 50% timing is later (Spring–Summer = before Julian Day (JD) 216, Summer–mid-Fall = after JD 216) (Supplementary Figs. 1, 2).

We carefully considered how to associate climatic data to our phenometrics before running models. While it remains possible that there are strong lag effects, where earlier climatic conditions have impacts on phenological sensitivity in a later part of the season, a logical first step is to use climatic conditions proximal to the phenological events being measured. We therefore opted to use Spring (March–mid June) summarized climatic conditions (e.g., average temperature, cumulative precipitation, and average vapor pressure deficit) for onset and summer/early Fall (late July—November) summarized climatic conditions for termination in initial model runs. While there is some variation in this timing across species, onsets and offset timing are within these seasonal time ranges or just after.

After assembling all the trait, climate and landscape data along with phenometrics, we fit linear mixed effects models (LMMs) with species as (intercept-only) random effects using the *Imer* package in R^45^. We are interested in interactions between climate, landscape and traits in these models but we avoided overly complex three-way interactions and cases where collinearity among predictors was damaging. We evaluated damaging collinearity using variance inflation factors (VIFs) generated by the ‘vif’ function in the R package *car*^46^, dropping two-way interactions that were highly collinear until all VIF scores were under 5. After fitting full models, we then used the function ‘step’ in the package *lmerTest*^47^ to select the best model after stepwise variable reduction. We determined model diagnostics for our best models using the R package *performance*^48^ and calculated marginal and conditional R^2^ values using the ‘r2_nakagawa’ function for mixed effects models^49^. We used the function ‘plot_model’ in the R package *sjPlot* to generate effects plots for key predictors^50^.

## Supplementary Information

Supplementary Information.

## Data Availability

Data and code used in these analyses are available through GitHub (https://github.com/Campbell-Lab-FMEL/mosquito-community-phenology).
