# Supplementary material for: Climate, landscape, and life history jointly predict multidecadal community mosquito phenology
Source: Sci Rep. 2023 Mar 8;13:3866. doi: 10.1038/s41598-023-30751-4 (PMC9995322; doi:10.1038/s41598-023-30751-4)
Supplement: Supplementary file 1 — Supplementary Information. [file 41598_2023_30751_MOESM1_ESM.pdf]

Supplementary Table 1. Onset, peak, and termination model results. Values include mean estimates followed by standard error values in parentheses. Values in bold indicate statistically significant variables determined with an alpha < 0.05.

Predictors	Onset	Termination	Peak
Intercept	**166.665 (6.371)**	**241.201 (4.985)**	**202.135 (4.364)**
Overwinter Eggs	7.881 (8.093)		
Overwinter Adult			
Spring-Summer Season			
Summer-mid-Fall Season	**35.434 (9.176)**	23.455 (9.273)	**42.042 (7.4508)**
Low Developed Landscape			
Medium Developed Landscape	**5.076 (2.495)**		4.285 (3.611)
Average Spring Temperature	-1.823 (1.537)		0.938 (2.570)
Average Fall Temperature		**6.539 (1.697)**	
Average Winter Temperature			
Average Spring Mean Vapor Pressure Deficit Index	**5.120 (1.941)**		**8.191 (3.433)**
Average Fall Mean Vapor Pressure Deficit Index		-1.649 (1.955)	
Average Winter Mean Vapor Pressure Deficit Index			
Average Spring Cumulative Precipitation	4.301 (2.355)		**8.460 (3.121)**
Average Fall Cumulative Precipitation		**4.418 (1.181)**	
Average Winter Cumulative Precipitation			
Summer-mid-Fall Season : Average Spring Cumulative Precipitation	3.761 (2.707)		
Summer-mid-Fall Season : Average Fall Temperature		**-5.910 (2.560)**	
Summer-mid-Fall Season : Medium Developed Land Cover Class	**9.715 (4.792)**		
Average Spring Cumulative Precipitation : Medium Developed Land Cover Class	**-4.475 (2.141)**		**-9.430 (3.625)**
Average Spring Cumulative Precipitation : Average Spring Temperature			**8.524 (1.7913)**
Average Spring Mean Vapor Pressure Index : Average Spring Temperature	**-2.292 (0.643)**		
Average Fall Mean Vapor Pressure Index : Average Fall Temperature		-2.027 (1.035)	
Overwinter Eggs : Average Spring Cumulative Precipitation	-3.525 (2.533)		
Conditional pseudeo R^2	0.59	0.499	0.391

Supplementary Table 2. Conditional values for intercept terms for species-level random effects for onset, peak, and termination model results.

Species	Onset	Peak Abundances	Termination
Aedes triseriatus	2.536	2.121	-4.387
Aedes trivattus	3.020	-1.859	-16.402
Aedes vexans	-5.556	-2.190	-0.276
Anopheles punctipennis	6.775	9.908	13.329
Culex pipiens	-6.775	-7.980	7.736
Culex territans	-7.567	-2.887	0.402
Uranotaenia sapphirine	7.567	2.887	-0.402

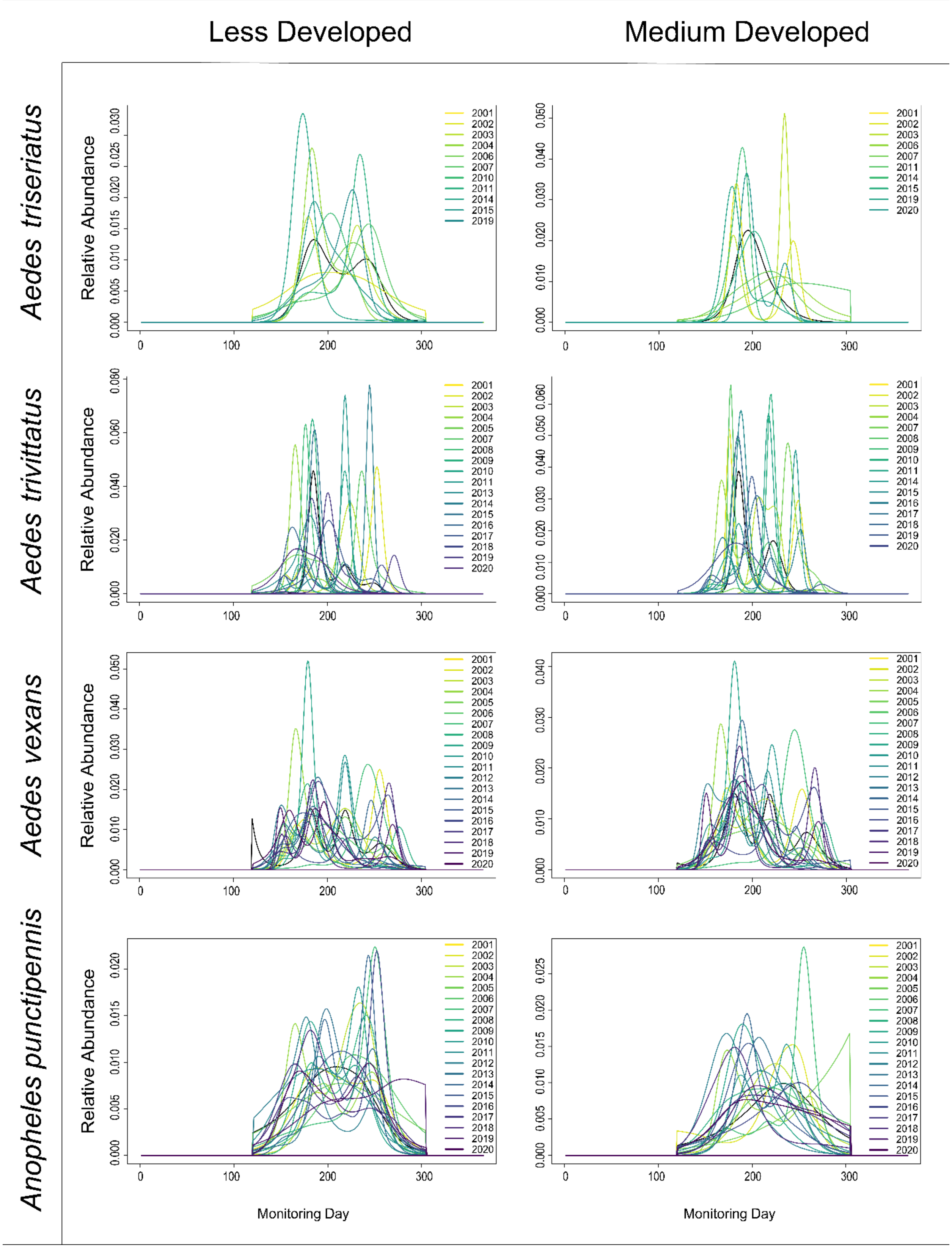

Less Developed
Medium Developed
Aedes triseriatus
Aedes trivittatus
Aedes vexans
Anopheles punctipennis
Relative Abundance
Monitoring Day
0
100
200
300
2001
2002
2003
2004
2005
2006
2007
2008
2009
2010
2011
2012
2013
2014
2015
2016
2017
2018
2019
2020

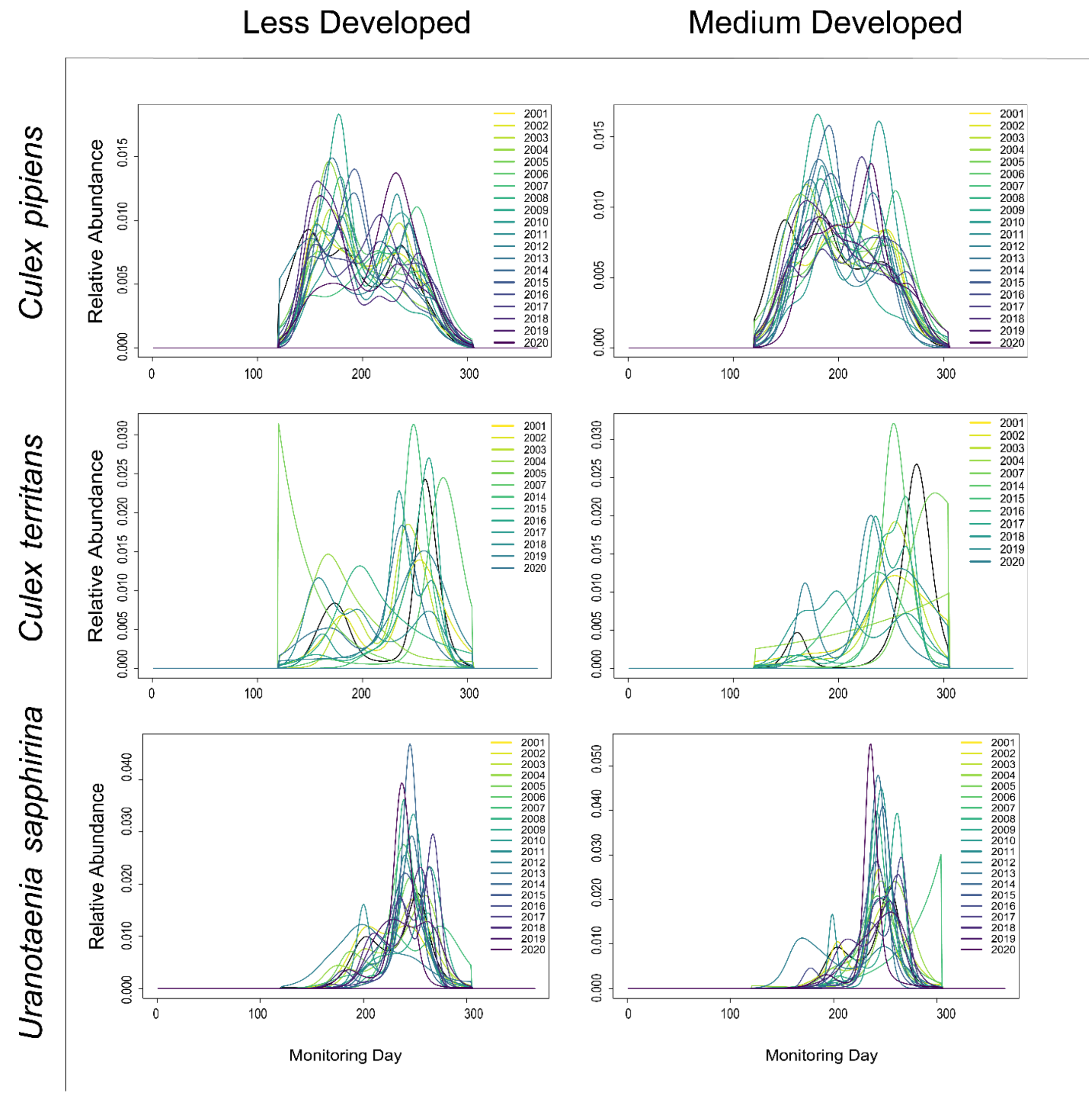

Supplementary Figures 1 & 2. Flight curves across less developed and medium developed landscapes for each of the seven species included in the study analyses. Flight curve plots were created using the ‘flight_curve’ function in the *rbms* package in R.

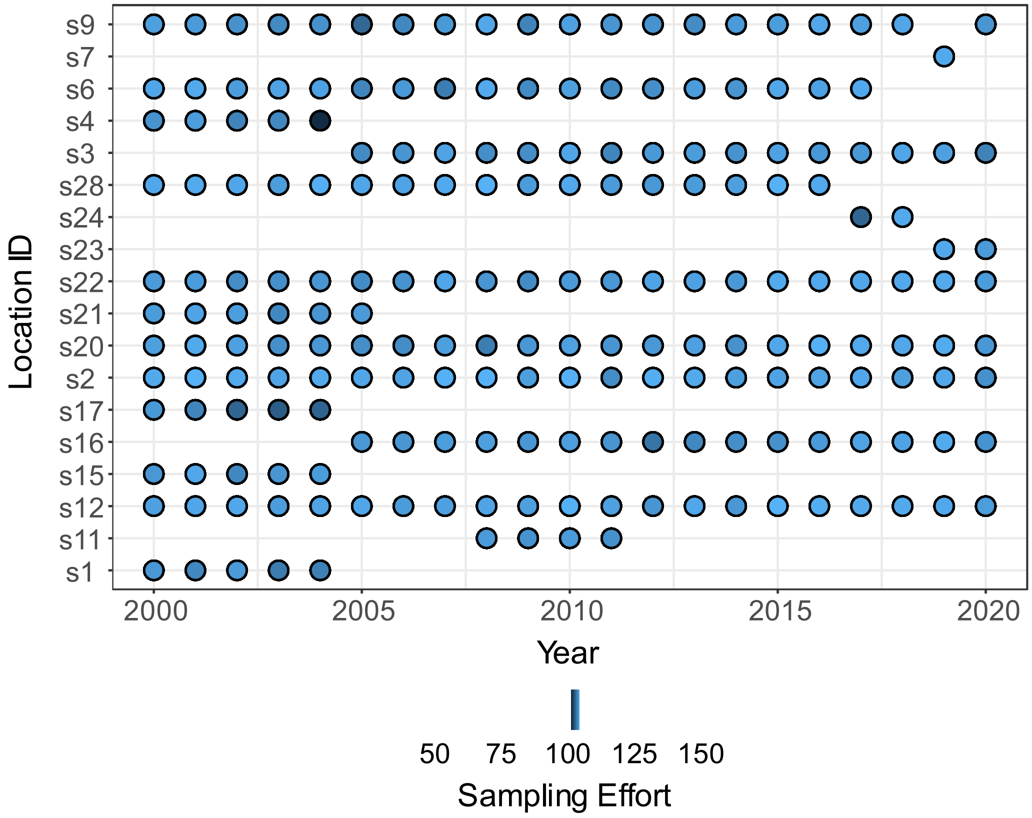

Supplementary Figure 3. Sampling distribution at trap locations between 2000 and 2020. Dark blue circles represent lower sampling effort in a given year and lighter blue circles represent greater sampling effort in a given year. Individual trap locations were classified as either less developed or medium developed land cover in each year and then phenometrics were then derived across trap locations corresponding to each land cover classification.
